# Points and Stripes: A Novel Technique for Masking Biological Motion Point-Light Stimuli

**DOI:** 10.3389/fpsyg.2018.01455

**Published:** 2018-08-28

**Authors:** Georg Layher, Heiko Neumann

**Affiliations:** Institute of Neural Information Processing, Ulm University, Ulm, Germany

**Keywords:** biological motion, articulated motion, point-light stimuli, perceptual salience, form, motion

## Abstract

Human articulated motion can be readily recognized robustly even from impoverished so-called point-light displays. Such sequence information is processed by separate visual processing channels recruiting different stages at low and intermediate levels of the cortical visual processing hierarchy. The different contributions that motion and form information make to form articulated, or biological, motion perception are still under investigation. Here we investigate experimentally whether and how specific spatio-temporal features, such as extrema in the motion energy or maximum limb expansion, indicated by the lateral and longitudinal extension, constrain the formation of the representations of articulated body motion. In order to isolate the relevant stimulus properties we suggest a novel masking technique, which allows to selectively impair the ankle information of the body configuration while keeping the motion of the point-light locations intact. Our results provide evidence that maxima in feature channel representations, e.g., the lateral or longitudinal extension, define elemental features to specify key poses of biological motion patterns. These findings provide support for models which aim at automatically building visual representations for the cortical processing of articulated motion by identifying temporally localized events in a continuous input stream.

## 1. Introduction

The investigation of human biological or articulated motion is still a major focus of scientific research. Deeper insights about the key structural features in space-time as well as the underlying computational principles have been revealed since the pioneering work of Johansson (1973). In his investigations of perceptual reference-systems in spatio-temporal cognition he proposed a method of using impoverished displays of biological motion stimuli. There, a set of lights move in coherence with the joints of a subject's skeleton (Johansson, 1973). Observers viewing animated sequences of such *point-light stimuli* (PLS) over a temporal period of 200 ms or longer are capable of reliably recognizing biological motions. Subjects are even able to discriminate different types of motions, such as e.g., *walking to the left, walking to the right* or *jumping jack* presented as PLS with stimulus onset intervals (SOI) of larger than 400 ms (Johansson, 1976). Over the last decades of more detailed analysis of the perceptual discriminative capabilities of human observers, the influences of global configurational and more local (or intermediate) level features have been investigated (Mather and Murdoch, 1994; Lange and Lappe, 2006; Garcia and Grossman, 2008). Apart from the impoverishment of the display, PLS contain *both* motion and—at least configurational—form information. This led to a continuing discussion about the relative contributions of these two visual input sources. The early processing stages of visual cortical processing of biological motion patterns is performed along two major processing streams, namely the ventral “*what”* and the dorsal “*where”* pathway. The two pathways later converge into fused representations at the level of the superior temporal sulcus (STS; Boussaoud et al., 1990; Felleman and Van Essen, 1991). Functional imaging studies (Grossman et al., 2000), as well as single-cell recordings (Oram and Perrett, 1994) indicate the existence of specific mechanisms for the processing of biological motion within this area STS. Further evidence suggests that cells in STS integrate form and motion representations of biological objects (Oram and Perrett, 1996) and selectively respond to face, limb, and whole body motion (Puce and Perrett, 2003).

These findings support model building about the functionality of segregated and interacting visual cortical transformations in biological motion perception. In particular, the modeling approach of Giese and Poggio (2003) suggests that the two parallel motion and form pathways process visual input largely independently over a hierarchy of stages incorporating homologous filtering and integration mechanisms in both pathways. As such, experimental as well as modeling investigations furthered the discussion about the contributions of form and motion information to the observed discrimination performance (Garcia and Grossman, 2008). This debate is still ongoing. Over the last decades, evidences from experimental studies indicated a strong influence of motion based features (Mather and Murdoch, 1994) and highlighted the importance of structural form information as well (Beintema and Lappe, 2002; Lange and Lappe, 2006; Hiris, 2007).

The experimental analysis of the contributions of form and motion information on the perception of biological motion sequences requires different visual parameters of motion and form, or shape, configurations to be isolated from each other. Taking the enormous variety of both motion and form based feature candidates into account, various studies identify different characteristics of low and high level visual cues which enable a reliable and robust recognition of biological motion sequences (Beintema and Lappe, 2002; Casile and Giese, 2005; Hiris, 2007; Thurman and Grossman, 2008; Thirkettle et al., 2009). Complementing a statistical analysis of form and motion contributions on biological motion recognition with a perceptual study, Casile and Giese (2005) conclude that patterns of local opponent motion (LOM) increase the perceptual salience and enable the robust recognition of a PLS. The investigations of Thurman and Grossman (2008) result in a similar conclusion highlighting the importance of LOM patterns by using “temporal bubbles” of point-light sequences embedded in additional noise dot motions. Beintema and Lappe (2002), on the other hand, show that PLS sequences can be robustly recognized without the presence of local image motion information. Further supporting the influence of spatio-configurational biological motion features, Hiris (2007) experimentally show that augmenting non-biological motion with structural information results in perceptual phenomena as reported similarly for biological motion stimuli. The work of Thirkettle et al. (2009) finally shows that the lateral extension of a biological motion PLS pattern correlates with the average human performance in discriminating such a pattern. Although the lateral extension of a PLS is solely defined by the spatial positions of the dots within a PLS pattern at a particular point in time (i.e., one articulated pose), it is is not free of temporal information when being related to the degree of lateral extension in the remaining sequence. Regarding biological motion as *deformation over time*, the maxima in form based feature channels might act as an anchor for characteristic form configurations (e.g., articulated poses). The distictiveness of such canonical representations, or key frames, might allow the reliable and robust recognition of the underlying biological motion.

Due to the properties of human kinetics, such maxima often co-occur with extrema in the motion energy of a displayed motion sequence. Thus, extrema in the motion energy might as well be candidates to act as a mediator indicating occurrences of form configurations with a high perceptual salience.

A key element in analyzing the relative contributions of form or motion based features on the perception of biological motion is the masking technique applied to isolate the feature under investigation and to suppress the immediate recognition of the displayed biological motion. One established way is to embed the spatio-temporal PLS pattern in a field of additional dots, either performing random motions or temporally incoherent motion patterns extracted from the biological motion PLS (e.g., Thurman and Grossman, 2008). The introduced dot motion noise aims at reducing the chance of perceiving the underlying skeleton structure as such and isolating relevant features at threshold level. A common critique of this masking paradigm argues that—particularly in case of random dot motion fields—the extraction of the embedded PLS is in fact facilitated by enabling figure-ground segregation. Focusing on the analysis of the contribution of local dot motion trajectories of a PLS, another class of masking techniques spatially scrambles the dot motions in PLS sequences (Grossman et al., 2000; Kim et al., 2015). This results in a dissolution of the spatial arrangement of the dot patterns and thus in a suppression of configurational form cues. Further PLS masking schemes aim at isolating specific stimulus characteristics by inverting the spatial arrangement (Sumi, 1984; Pavlova and Sokolov, 2000; Troje and Westhoff, 2006) or modifying the number / locations of visible dots anchored on the underlying body skeleton (Dittrich, 1993; Beintema and Lappe, 2002).

In the present contribution we propose a different set of visual stimuli which capture biological motion in PLS but utilize complex patterns of form as overlays radiating from an external reference point that serves as center location for the dynamic form pattern. The main aim of the investigation is to justify the hypothesis derived from a previous modeling investigation (Layher et al., 2014), that articulated motion sequences are segmented by key poses on the basis of specific form defined cues (high degree of articulation). There, events of distinguishing shape characteristics are identified by analyzing simple motion based features (extrema in the motion energy). Thus, the motion information mediates the salience of the form pattern with respect to the biological motion sequence. It has been demonstrated that such key poses capture highly informative characteristics about poses and actions that allow to already classify activity components by combined form and motion information (Layher et al., 2017). We conjecture that points of maximum limb expansion identify such key poses within biological motion sequences and are correlated to extrema in basic feature parameters, e.g., local maxima in the overall motion energy. In order to isolate the parameters under discussion and degrade additional supporting features, such as a priori knowledge about the type of motion, the recognition of the underlying skeleton, or symmetry effects, we propose a novel masking technique, applying an overlay pattern on the PLS. The temporally varying center point of the dynamic pattern is randomly defined in a spatial neighborhood of the center of gravity of the PLS, while the different lines which radiate from the center point are connected to each of the point-lights in the target stimulus. Together, the dynamic radial line pattern defines a new masking structure which inherits part of the articulated motion but transfers this into the overlay instead of the PLS. Details of the stimulus generation and its structure are discussed in sections 2.2, 2.2.2 and in **Figure 2**. The main motivation of using this new kind of stimuli is that the motion and configural information of the PLS is still intact but the form encoded by the ankle configurations is severely impaired in its visibility through the dominating dynamic radial line patterns that define the mask. Opposed to varying masking techniques, the proposed stimuli prevent the visual system from potentially segregating the PLS from the masking noise stimuli in a figure-ground separation step. Using such stimuli we ran four different experiments to judge whether subjects can rapidly recognize a masked *jumping jack* PLS sequence and to analyze the influence of the lateral and longitudinal extension on the measured human recognition performance. In addition, we analyzed manipulated versions of the masked PLS, which are generated by removing the turning in the motion of the left and the right hands and by temporally shifting the motions of the left body parts.

Our results demonstrate and confirm that characteristic properties of form features define elemental features to specify the occurrence of key configurations, or poses, of biological motion patterns as they occur in natural articulation sequences. Such maxima go synchronously with local extrema in motion energy, as represented in the dorsal pathway of the visual system. Our findings indicate that maxima in form based feature channels (e.g., the lateral and longitudinal extension) do not solely determine the identification of key poses but serve as tags to be further utilized in conjunction with localized temporal events. The identification of such multidimensional feature selection mechanisms provides support for modeling investigations to automatically build visual representations based on key poses in the cortical processing hierarchy of articulated motion.

The correlation between the lateral extension and the perceptual saliency of a biological motion subsequence shown by Thirkettle et al. (2009) for a PLS displaying a walking gait could successfully be reproduced for a jumping jack sequence when further reducing the visual cues in the PLS through the proposed masking technique and even when the human observers are not able to recognize the motion underlying the dot motion pattern.

The rest of the paper is structured as follows. Section 2 describes the acquisition of the motion capture data for the generation of the PLS and further details the creation of the PLS variations, the masking technique, as well as the experimental design and setup. In section 3, the the results of the conducted experiments are presented and interpredted. In the last section 4, the implications of the conclusions drawn from the observed measurements are discussed.

## 2. Methods

### 2.1. Motion capture data

Stimuli were generated using data obtained from the *uulm multiperspective action recognition dataset* (uulmMAD^1^) which contains several sets of synchronized motion capture (MoCap) and video sequences. The dataset consists of 31 subjects performing 14 actions from activities of fitness/stretching and everyday life. The subjects' skeletons were recorded using an inertial motion capturing system (XSens MVN Biomech) operating at a sampling rate of 120 Hz (Glodek et al., 2015). Out of the 23 captured skeleton points, a total number of 18 were selected to constitute the PLS (displayed in solid black in the first column of **Figure 2**). The motion capture data of 20 subjects, each performing four repetitions (cycles) of a jumping jack action in three separate recordings, was first normalized in speed (resulting in a final duration of one jumping jack cycle of 150 samples/1.25 s), orientation (0° hip rotation) and height (with a longitudinal distance between the head and the left toe of 180 cm in a neutral pose) per person and then averaged over all subjects. The final stimulus consisted of 1.5 jumping jack cycles (225 samples/1.875 s) and was subsampled to match a temporal resolution of 100 Hz using shape-preserving piecewise cubic interpolation (resulting in 187 frames). The number of cycles was chosen such that the final sequence not only contains a full cycle of the jumping jack but additionally covers all moments of direction reversal in their temporal context.

### 2.2. Generations of point-light stimulus variations

#### 2.2.1. Sequences with phase variations

Three variations of the jumping jack PLS were generated for the different experimental configurations. Under all conditions, the 3D motion capture data was first projected onto the image plane by orthographic projection. Motion sequences were generated for three different temporal pattern configurations, namely, a sequence displaying the unmodified PLS jumping jack (*unmodified*), a modified version that contains loopy articulated limb extensions (*looped*), and a shifted counter-balanced articulation sequence (*shifted*; see **Figure 2**).

The *unmodified* version directly uses the sequence of point positions derived from the motion capture data as described in section 2.1.

In the *looped* configuration, the lateral component of the hands' motion direction is inverted whenever one of the hands changes the direction of motion. Thus, the resulting motion pattern of the hands does not contain any reversal points and the lateral extension is reduced in the mid part of the sequence. The transformation is realized by mirroring the position of the left and right hand around the axis through the left, respectively the right elbow, parallel to the ordinate of the image plane. As a result of the transformation, the hands perform two counter-rotating circular motion patterns (loops). The key rationale for introducing such variations in the unmodified PL displays is to selectively manipulate appearances in the overall body movements. Thus the signature of upper body and arm movements is significantly varied: (a) changes in the limb movement direction are eliminated (*looped*) and (b) the symmetry of the limbs' movements is eliminated (*shifted*). Equal to the *unmodified* sequence, the *looped* sequence consists of 187 frames.

**Figure 1:**
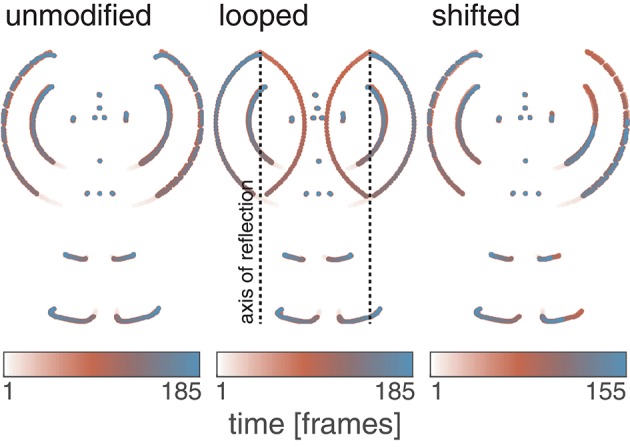
Simulus composition. Three different variations of point-light stimuli were derived from an average normalized motion capture sequence recorded for 18 subjects performing the action of *jumping jack*. In the *unmodified* version, the projections of the 3D motion capture points were directly used to build the stimulus. In the *looped* variant, the horizontal component of the hand positions are transformed such that the hands perform two counter-rotating circular motion patterns. The *shifted* variation was obtained by temporally shifting the point positions of the left body half by 30 frames and results in a minimal variance of the observed lateral extension of the dot pattern. Time is displayed color-encoded from white (first frame) through red to blue (last frame). Note the asymmetry of the left and the right body half for the *shifted* version.

The *shifted* variation aims at reducing the variance in the lateral extension of the stimulus without affecting the longitudinal extension or the motion patterns of the individual points. This is achieved by temporally shifting the motions of the left body parts such that changes in the longitudinal extension caused by motion of right body parts are counterbalanced. The maximum reduction was obtained for a temporal shift of 30 frames (0.3 s), decreasing the standard variation of the observed lateral extension by a factor of 3.8. Compared to the *unmodified* and *looped* sequences, the final number of the frames in the *shifted* configurations is reduced to 157 frames. Figure 1 visualizes the trajectories of the point-lights for the three stimulus configurations using color encoding. In contrast to the *umodified* sequence, the hands show a circular trajectory for the *looped* variant, while the *shifted* sequence possesses an asymmetrical motion pattern.

#### 2.2.2. Masking

**Figure 2:**
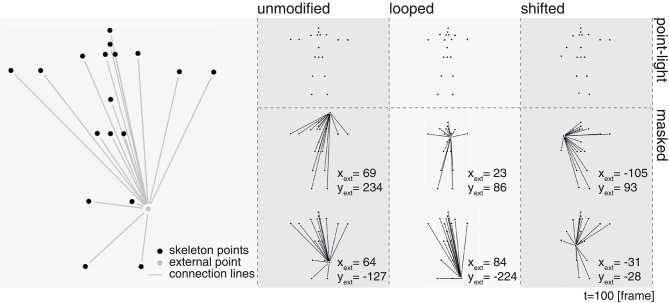
Masking of biological motion point-light simuli. In order to mask the underlying PLS sequence, each of the skeleton points was connected to a randomly drawn static external point. On the left, the skeleton points (black) in one frame are shown alongside with an exemplary external point and the connected radial lines (displayed in gray, the final stimulus was rendered with all points and lines in white on a black background). From the second to the forth column, static snapshots of the unmasked point-light stimuli (first row) and their masked counterparts for two different external points (second and third row) are displayed for the three variations of the jumping jack PLS sequence.

The displays utilized here each consist of PLS combined with dynamic radial line patterns such that the stimulus appearance leads to masked spatio-temporal motion sequences (see Figure 2). In a “marionette-like” manner, the spatial patterns are centered to a randomly drawn fixed reference point around the central part of the display and spread toward the points of the PLS dot pattern. Our goal is to identify the relative importance of specific poses during a displayed PLS articulation sequence. We argue that in previous stimulus configurations which have used overlaid spatio-temporal dots as noise patterns one cannot exclude the possible conclusion that an observer is able to segregate the moving PLS from the noise pattern and then solve the given task of, e.g., deciding the walking direction or classifying the articulated motion pattern. The stimuli used here, on the other hand, should be immune to mechanisms of figure-ground segregation. The stimulus strength of the apparent form of the line pattern dominates the global configural appearance of the articulation. Since the external point that serves as the center for the radial line bundle pattern is randomly generated, subjects in the experiment cannot adapt to a spatially symmetric dynamic form for improving the judgment of the point-light motion pattern.

### 2.3. Stimulus display

Stimuli were rendered using Matlab 2015b in conjunction with the Psychtoolbox 3 (Brainard, 1997; Kleiner et al., 2007)^2^ on an Acer XB270HU monitor with a display diagonal of 27 inches, a resolution of 2, 560 × 1, 440 px and a maximum refresh rate of 144 Hz. A constant distance of 60 cm between the subjects and the screen was ensured using a chin rest. Each of the point-light dots was rendered at a size of 12 arcmin. In case of the masked stimuli, the position of the additional static point was randomly drawn from the range of the maximum bounding box enclosing all original point positions of the original stimulus (approximately 7.82° lateral and 10.60° longitudinal viewing angle) using a uniform distribution. The width of the line interconnecting a point-light dot with the external static dot was set to 2 px. The lines do not fully connect a pair of two points, but end when a 5 px distance to the contour of a point is reached. The point-light stimuli were always presented with the hip point fixed at the center of the display and the frontal plane orthogonal to the viewpoint of the observer. Figure 2 shows examples of the generated stimuli for both, the original PLS display of the jumping jack (point-light), as well as the configuration with the PLS points connected to two different exemplary static external points (masked) for frame 100. Note how the two different external points change the appearance of the stimulus, although the positions of the remaining point-lights are the equivalent.

The lateral and longitudinal extension of a point-light pattern is determined by calculating the width and height of the bounding box enclosing all point-lights within one frame. A superposition of the extension in the two directions is calculated by directly summing the two values, logically acting as an “*or*” operation. The resulting values are normalized by dividing through the maximum extension (lateral and longitudinal) over the whole PLS sequence. Equally normalizing the integrated L2-Norm of the point-light motion vectors between two adjacent frames finally gives the relative motion energy.

### 2.4. Procedure

Since the main focus of the presented study was to analyze the effectiveness of the proposed masking scheme and to allow for a direct comparison, we closely followed the experimental paradigm described in Thirkettle et al. (2009) by employing the same temporal two-alternative forced choice (2AFC) task and adopting the reported threshold duration of 50 ms. The three PLS sequence variations were subdivided in non-overlapping temporal windows of 50 ms length (5 frames), resulting in 37 subsequences for the *unmodified* and the *looped* sequence, as well as 31 for the *shifted* stimulus variation. For each stimulus presentation, a different external static point was selected randomly.

A second sequence of the same length was derived by temporally phase-scrambling the motion patterns of the skeletal points represented in the PLS. This was achieved by randomly drawing an individual temporal starting point for each of the points (from a uniform distribution) and merging the respective 50 ms sequences into one (now incoherent) motion pattern. The same external point was used for the masking of the phase-intact and the phase-scrambled incoherent dot motion patterns. The two patterns were presented in a random temporal order. For each stimulus pair, participants had to decide which of the two 50 ms subsequences was extracted from the original sequence. After the experiment, the subjects' performance in separating the phase-scrambled control from the phase-intact pattern is used as an indicator for the perceptual salience of the particular subsequence.

Since the masking of the dot patterns through the connecting lines results in a different visual appearance of each stimulus, the complete phase-intact masked PLS sequence was presented prior to the display of the 50 ms phase-intact subsequence and the phase-scrambled control. A fixation cross was displayed for a duration of 1 s before and after the presentation of the full sequence and a blank screen was shown for 1 s between the phase-intact test and the phase-scrambled control pattern. The resulting total presentation time per stimulus condition was 4,950 ms for the *unmodified* and the *looped* variations, and 4,650 ms for the *shifted* PLS sequence. All stimuli configurations were presented twice per subject and in randomized order. Examples of the four stimulus variations are included as video sequences in the supplement to this contribution (100 fps playback speed; Supplementary Videos).

The experimental procedure was introduced to the participants by providing an explanation of the display setup and the task. However, no details were given in any explanation about the background of the experimental question and in particular terms like “biological motion,” “human,” or “action” were avoided. Subjects were solely instructed to decide whether the first or the second of the two presented dot motion patterns was a subsequence of the PLS sequence displayed before. After the completion of an experimental session, each subject was asked to provide a description of the nature of the dot motion patterns they observed during the experiment using a questionnaire.

### 2.5. Participants

Forty-one healthy naïve subjects (average age 27.46, std 4.40) with normal or corrected to normal vision participated in the experiment. The *unmodified* stimulus configurations were presented in random order together with the *looped* variations (see Figure 2) to a group of 21 subjects. A second group of 20 subjects performed the experiment with the same *unmodified* stimulus configurations, but in addition with the *shifted* variations presented in random order as well. After a short break, ten out of the 20 subjects additionally performed the same experiment but with unmasked point-light stimuli (see Figure 2). In doing so, we aimed at reproducing the results reported for the unmasked PLS display of a jumping jack in Thirkettle et al. (2009). The presented study was conducted according to the ethical guidelines set out in the WMA Declaration of Helsinki (ethical committee approval was granted: 196/10-UBB/bal) with written consent from all subjects. The study protocol was approved by the ethics committee of the University of Ulm^3^.

## 3. Results

### 3.1. Experiment I

Thirkettle et al. (2009) observed a strong correlation between the lateral extension of a PLS pattern and the average human performance in discriminating 50 ms phase-intact sequences from phase-scrambled controls using a PLS display of a human walking gait. In a variation of the experiment, Thirkettle et al. (2009) replaced the walker with a PLS display of a jumping jack sequence. Contrary to the results obtained for the walker, no correlation between the lateral extension of the PLS and the detection performance was observed. A potential explanation for this degradation is given by the strong symmetry of the point arrangement in the motion pattern which is corrupted in the temporally scrambled stimulus and thus an easy recognition of the target stimulus is possible.

**Figure 3:**
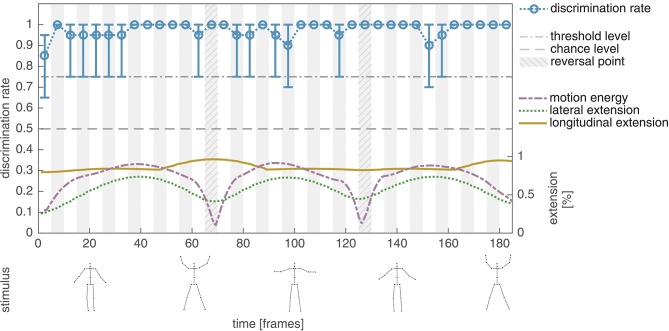
Experiment I, average discrimination performance for the unmodified point-light jumping jack sequence. The average discrimination rate of ten subjects is plotted together with the 95% confidence interval derived by applying bias corrected and accelerated percentile bootstrapping with 100,000 bootstrapping samples (blue). In addition, the normalized lateral (green) and longitudinal (yellow) extent of the minimal bounding box enclosing all points of the PLS per frame. In the bottom row, exemplary snapshots of the stimuli are shown. For a better traceability, point-lights are additionally connected by lines indicating the underlying skeleton. The skeleton lines are not shown in the displays of the experiments. As reported in Thirkettle et al. (2009), the average discrimination rates show no correlation to the lateral extension of the unmasked PLS for a jumping jack sequence.

In the first experiment of the present study, we aim at replicating the results reported in Thirkettle et al. (2009) by using an unmasked unmodified jumping jack point-light sequence following the described experimental paradigm as close as possible. Figure 3 shows the average discrimination rates of ten naïve subjects for the unmodified point-light configuration without masking. Independent of the temporal window the discrimination rate is almost constant at ceiling level around 0.98 (mean: 0.98, std: 0.04). As reported in Thirkettle et al. (2009), the discrimination of the phase-intact motion pattern from the phase-scrambled control is no difficulty for unmasked PLS and thus no correlation to the normalized lateral extension of the stimuli can be revealed (*r* = 0.10, *p* > 0.5).

After the conduction of the experiment all participants were able to directly identify the observed unmasked dot motion pattern as a display of a human performing the action *jumping jack*.

### 3.2. Experiment II

**Figure 4:**
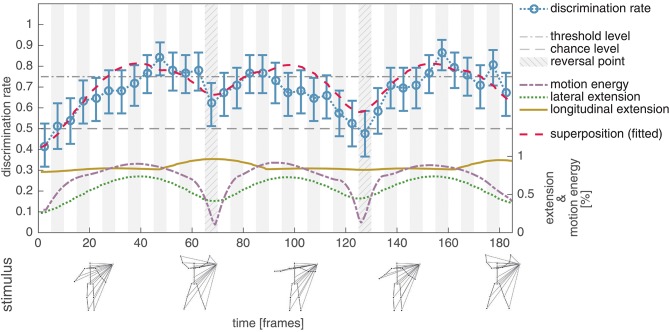
Experiment II, average discrimination rate for the unmodified masked jumping jack sequence. The average discrimination rate of 41 subjects is plotted together with the bootstrapped 95% confidence interval (blue, compare Figure 3). In contrast to the unmodified PLS sequence in Experiment I, the average discrimination rate now shows a correlation to the lateral extension (dotted green) of the masked sequence (*r* = 0.67, *p* < 0.001). The discrimination rate for the subsequence covering frames 66–70 is clearly increased compared to frames 126–130, although the lateral extension is at the same level. This might indicate, that next to the lateral extent, the longitudinal extent (solid yellow) also contributes to the perceptual salience of the stimulus subsequence. This hypothesis is further supported by a strong correlation between the superposition of the lateral and longitudinal extent (dashed red) with the average detection performance (*r* = 0.83, *p* < 0.001).

In the second experiment the dot motion patterns from Experiment I were masked by lines connecting the points in the PLS to an additional external point (see section 2.2.2 for a detailed description of the stimulus configuration and the logic behind). As expected, this further reduces the visual cues in the PLS and results in a decreased average discrimination rate (mean: 0.68, std: 0.10). However, in contrast to Experiment I, it is now possible to observe a correlation between the lateral extension of the PLS dot pattern and the discrimination performance (*r* = 0.67, *p* < 0.001). Figure 4 shows the average discrimination performance, alongside with the relative lateral and longitudinal extension of the PLS display, as well as the relative amount of motion energy.

The displayed results indicate that, next to the lateral extension, the longitudinal extension also influences the recognizability of a PLS subsequence (thus implicating an increase of the perceptual salience). In particular, the two subsequences covering frames 66–70 and 126–130 are almost equal in their normalized lateral extension (averages of 0.42 and 0.46), but differ in the average of their normalized longitudinal spread (0.96 vs. 0.82). The measured human discrimination performances reflect this difference between the two subsequences (mean: 0.62, std: 0.13 for starting frame 66; mean: 0.48, std: 0.06 for starting frame 126). Correlating a measure combining the lateral and longitudinal extent by superposition (*r* = 0.83, *p* < 0.001) further supports the conjecture that, alongside the lateral extension, the longitudinal extension might increase the perceptual salience of a subsequence.

After the experiment, about two thirds (68.29%) of the participants were not able to name the kind of motion underlying the presented stimuli in the subsequent questionnaire. The remaining third (31.71%) characterized the sequences as biological or human motion. Note that the described effects were observed although all of the stimuli had a different visual appearance and the majority of the participants did not recognize the motions as biological. Compared to unmasked PLS stimuli, or an embedding in random dot motion patterns, the proposed masking thus reduces the chance of an influence on the discrimination rate of high level processes, such as skeleton fitting or the utilization of a priori knowledge about the kind of action displayed in the PLS sequence. Together, these results indicate, that by applying the described masking technique, the effect reported in Thirkettle et al. (2009) can also be observed for a jumping jack sequence after reducing visual cues and that the increased perceptual salience of point patterns with a large spread is not dependent on the direct perception of a biological motion PLS as such.

### 3.3. Experiment III

**Figure 5:**
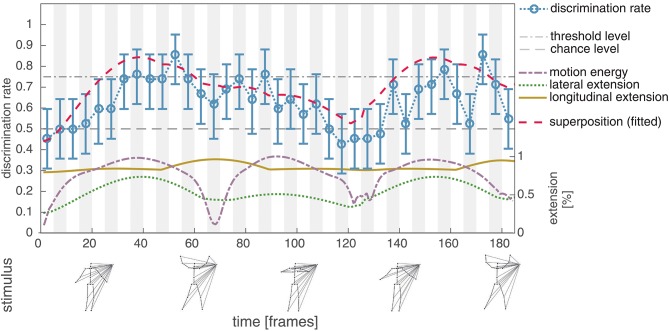
Experiment III, average discrimination rate for the looped masked jumping jack sequence. The average discrimination rate of 21 subjects is plotted together with the bootstrapped 95% confidence interval (blue, compare Figure 4). As in Experiment II, the discrimination rate is strongly correlated with the combined (dashed red) lateral (dotted green) and longitudinal (solid yellow) extension (*r* = 0.71, *p* < 0.001). Removing the turning points of the hand does not have an effect on the measured discrimination rates, whereas the decreased lateral extension slightly reduces the discrimination performance.

In the third experiment we modified the PLS sequence such that the hands as part of the most extendable body configuration follow a counter-rotating circular movement, while the remaining body parts perform the unaltered jumping jack (see Figure 1; for details refer to section 2.2). This modification has two major effects on the resulting stimuli. First, the lateral extension is reduced during the middle part of the sequence. Second, due to the counter-rotating motion pattern of the hands the occurrence of LOM patterns is increased within this region. For the *looped* stimuli, participants achieved an average discrimination rate similar to Experiment II (mean: 0.63, std: 0.12). As in Experiment II, a correlation between the human discrimination performance and the combined lateral and longitudinal extension can be observed for the *looped* stimuli (*r* = 0.71, *p* < 0.001; see Figure 5). The discrimination rate for the modified parts of the sequence (subsequences with starting frames ∈ [66, …, 126]), however, is slightly decreased (mean: 0.60, std: 0.13) when compared to the *unmodified* stimuli (mean: 0.65, std: 0.10). A direct effect on the discrimination performance measured for subsequences with starting frame 66 (mean: 0.62, std: 0.13 for *unmodified*; mean: 0.62, std: 0.08 for *looped*) and 126 (mean: 0.48, std: 0.06 for *unmodified*; mean: 0.45, std: 0.08 for *looped*) caused by the missing turn in direction of the hands cannot be observed.

Notably, the discrimination rate in between starting frames 66 and 131 decreases only marginally compared to the performance measured in Experiment II although the lateral extension within the subsequence is clearly decreased. Possibly, the characteristic local (opponent) motion patterns introduced by the counter-rotating circular motion of the hands support the discrimination of the phase-intact target and phase-scrambled control. Again, this might be an indication, that the perceptual salience of a subsequence is not driven by characteristic occurrences (e.g., local maxima) in one single, but a variety of feature channels.

### 3.4. Experiment IV

**Figure 6:**
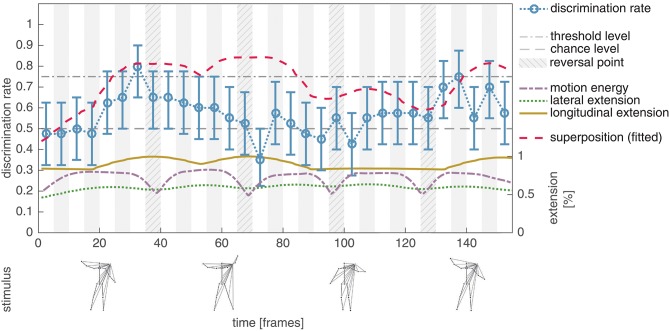
Experiment IV, average discrimination rate for the shifted masked jumping jack sequence. The average discrimination rate of 20 subjects is plotted together with the bootstrapped 95% confidence interval (blue, compare Figure 4). Compared to the results of Experiment II, the average discrimination rate is decreased (mean: 0.57, std: 0.12). A high discrimination performance tends to co-occur with an increased longitudinal extension (solid yellow), with exception of the subsequences starting with frames 61, 65, and 71. This results in a decreased correlation between the combined extension feature (dashed red) and the discrimination performance, which might be explained by the statistics of the asymmetric dot patterns within the PLS sequence (see text for details). Nevertheless the results indicate that although there is just little variance in the lateral extension (dotted green) of the PLS, there are distinct events with an increased perceptual salience.

Experiment IV aimed at systematically reducing the variance of the lateral extension over the whole jumping jack point-light sequence without modifying the local motion patterns of the individual dots (*shifted* stimulus configuration; see section 2.2 for details). In addition, this modification implicitly prevents the occurrence of LOM patterns caused by the hands. By temporally shifting the motions of the left body parts, the standard deviation of the lateral extension was reduced by a factor of 3.8 from 59.71 px to 15.66 px. This results in a decreased average discrimination rate (mean: 0.57, std: 0.12) when compared to the measurements for the *unmodified* stimulus configuration (mean: 0.68, std: 0.10) and thus supports the assumed impact of the lateral extension on the human discrimination performance. The second hypothesis underlying Experiment IV was that decreasing the variance of the lateral extension results in a clearly observable—since isolated—influence of the longitudinal extension on the perceptual salience of a subsequence, as well as a correlation of the combined extension values to the discrimination performance similar to Experiment II and III. Correlating the summed lateral and longitudinal extension to the discrimination performance (*r* = 0.31, *p* = 0.08), which is dominantly driven by the contribution of the longitudinal extension (*r* = 0.33, *p* = 0.07), does not directly support this hypothesis. In Figure 6, a tendency of the discrimination performance to increase with the longitudinal extension of the stimulus can be observed, but with the exception of the subsequences with starting frames ∈ [61, …, 76].

Noticeably, the discrimination performance clearly falls below chance level (mean: 0.35, std: 0.08) for the subsequence with starting frame 71, indicating a systematic articulated motion discrimination. In all, these mistake by the subjects in choosing the non phase-scrambled point-light pattern. A possible explanation of this effect might be the asymmetric nature of the point configurations and the statistics of the asymmetric patterns within the whole PLS sequence. Subjects might perceive the configuration displayed from frame 71 to 75 as a deviation from the subsequence starting with frame 41 and as a consequence tend to select the phase-scrambled PLS pattern. This assumption—if applicable—might explain the only fragmentary correlation between the longitudinal extension and the discrimination performance displayed in Figure 6, but is a topic that needs to be further analyzed experimentally.

Nevertheless, the results obtained in Experiment IV show that even though the lateral extension is almost constant over the displayed PLS sequence, there are distinct moments in time (events) with an increased discrimination rate (indicating a higher perceptual salience). These events are partially co-located with increasing values of the longitudinal extension but given the obtained results we cannot exclude that another feature might be causally responsible for this effect.

## 4. Discussion

### 4.1. Articulated motion, perceived pattern, and its masking

The perception of articulated motion, or biological motion, is still a target of detailed investigation. Perceptual degradations have been studied to reveal the contributions of different representations and dynamic mechanisms in visual cortex which contribute to such coherent form-in-motion percepts. In this contribution we introduced a novel PLS masking approach, which allows to selectively impair the ankle information of the body configuration while keeping the motion of point-light locations intact. The mask is centered at a (randomly selected) anker point with radial line connections from this point to each joint represented in the PLS. The line endings leave a small distance to the individual point-light in order to leave the original PLS items unimpaired. The rationale to employ this new configuration is to counteract possible spatio-temporal groupings in the PL walker configurations. Coherently moving dots can give rise to contour grouping effects, following the Gestalt laws of proximity and common fate (Metzger, 1936; Stevens, 1978; Metzger, 2006; Thirkettle et al., 2009; Wertheimer, 2012). The neural mechanisms underlying such grouping phenomena have been explored to a certain degree but remain elusive when complex dynamic item configurations are displayed. Inspired by Ullman (1984), Roelfsema distinguished different grouping mechanisms, namely, base-grouping and incremental grouping, respectively, the first operating automatically and fast utilizing primary features, while the latter dynamically links multiple features flexibly taking more time (Roelfsema, 2006). Experimental evidence suggests that feature-based base-groupings are encoded in the feedforward sweep of cortical processing while incremental encodings are mediated by horizontal and feedback processing utilizing knowledge and contextual information to modulate lower-level neural representations (compare Phillips et al., 2015).

The radial line mask pattern proposed in this study comprises a dynamic physical luminance pattern that strongly overcasts any dynamic shape configurations imposed by the dynamic point patterns. In order to leave the configuration of the PLS itself unimpaired we left the line endings disconnected to the individual dots. Our reasoning is that any groupings which might be dynamically established to segregate spatio-temporal figural patterns from background (Sporns et al., 1991) will operate incrementally and, thus, on a slower time-scale. Such line mask pattern will be immediately visible and the spatio-temporal effects created by such masks are mainly determined by base-groupings. Based on such segregation of different temporal phases of neuronal processing, we believe that the individual studies conducted in the reported experiments allow us to dissect the contribution of different stimulus elements in the perceptual judgement of biological motion. While we are investigating the selective manipulation of articulated limb trajectories - represented in the still intact PLS—the radial line mask reflects the primary pattern but suppresses secondary grouping operating on slow temporal scales.

### 4.2. Experimental investigation of articulation patterns and their temporal structure

Following the experimental methodology and paradigm employed in Thirkettle et al. (2009), we studied the effect of three masked PLS sequence variations on the measured performance in discriminating a phase-intact target PLS subsequence and a phase-scrambled control. In line with (Thirkettle et al., 2009), the results show that the lateral extension of a PLS pattern correlates with the average discrimination performance and thus is a candidate to act as a predictor of the perceptual salience of specific moments in the course of articulated motion sequences. In addition, the reported results show indications, that the longitudinal extension of a point-light pattern might as well support the perceptual salience of a PLS subsequence (*unmodified*). Decreasing the variance of the lateral extension by modifying the motion trajectory of the hands alone only resulted in a slight decrease of the discrimination rate, thus opening the question whether the implicitly introduced local (*opponent*) motion patterns might be responsible for an increased perceptual salience (*looped*). In a further experiment, the PLS sequence was modified, such that the lateral extension was almost constant and the occurrence of LOM pattern was excluded (*shifted*). The measured discrimination performance was clearly decreased, which suggests less importance of flow contrast detection as specific feature for articulated motion discrimination.

In all, these results indicate, that first order changes in the representations along characteristic feature channels, such as e.g., extrema in the lateral extension of the body configuration during an articulation, might act as triggers or mediators for the establishment of key representations used to identify PLS sequences (Nothdurft, 2000a,b).

In Experiment I, we first reproduced the results reported in Thirkettle et al. (2009) for separating 50 ms subsequences of an unmasked PLS displaying a jumping jack from phase-scrambled control patterns in a temporal 2AFC task (Experiment I; section 3.1). In both displays, the discrimination performance saturated almost at ceiling. In contrast, the results obtained in Thirkettle et al. (2009) for a PLS sequence displaying a human walking gait showed a direct correlation between the measured task performance and the lateral extension of the point-light pattern. Thirkettle et al. (2009) propose that the constant task performance found for the jumping jack PLS sequence might possibly be explained by the exploitation of symmetry properties of the point-light configurations and the detection of violations thereof. Furthermore, the authors argued that the measured performance is strongly dependent on the displayed action, as well as the experimental task and methodology (e.g., the kind of noise, which is applied on the stimulus).

Accordingly, Thurman and Grossman (2008) obtained different results for experimentally analyzing the perceptual salience of subsequences of a jumping jack PLS using a different methodology. There, subsequences with different starting frames were embedded in a field of noise dot motions at randomized spatial positions with trajectories drawn from the original sequence but with individual starting frames. The target dot motion patterns emerged as “temporal bubbles” within random dot motion sequences and subjects were asked to discriminate sequences containing a target pattern from scrambled motion controls in a threshold task. Experiments were conducted to identify subsequences containing key features which drive an increased perceptual salience. For a jumping jack, as well as walking gait PLS sequence, the measured task performance clearly indicated that patterns of opponent motion might constitute such a key feature (supporting the role of opponent motion as a feature to perceive biological motion; Casile and Giese, 2005). Opponent motion is most pronounced when the relative distance between the arms and the legs is smallest. This result is—in case of the walking gait PLS—in conflict with the results reported in Thirkettle et al. (2009), where subsequences containing highly articulated postures with a large lateral extension were identified as perceptually more salient. As discussed in Thirkettle et al. (2009), these contradictory results might be explained by low-level effects caused either by the density of the noise dots used for the masking in case of a threshold task, or, on the contrary, by the modified spread of dots in discriminating a target pattern from a phase-scrambled control.

In our study in Experiment II, we applied the masking technique as described in section 2.2.2. As a result of reducing the visual cues we were able to show a correlation between the extent of a jumping jack PLS pattern and the performance in discriminating it from a phase-scrambled control. The same relationship was reported in Thirkettle et al. (2009) for an unmasked walking PLS, but could not be replicated for an unmasked jumping jack PLS. Although we cannot guarantee that the proposed new masking method excludes the influence of low-level cues introduced by the phase-scrambling, we argue that this effect is at least weakened. This is likely since the visual appearance of the biological motion pattern is altered for each individual stimulus by the randomly positioned additional external point. Introducing the mask pattern reduces the chances that fast base-groupings are established which provide the input to higher-level processes, such as e.g., skeleton fitting. Even more, the potential utilization of prior knowledge about the kind of action pattern that guides incremental groupings is diminished. The semantics of the motion signature is extinguished as indicated by the separate questionnaire to identify the pattern (after the experiment only one third (31.71%) of the subjects was able to identify the observed masked PLS as a biological or human motion). Local motion sub-patterns, such as LOM, positioned at locations internal to the figural pattern are suppressed as well. Temporal maxima in the whole body appearance here co-occur with maxima in feature channel activations detected by observers. Our experimental procedure thus enables segregating the perceptual influences of extremal global feature properties in global configural appearances from local salient features such as LOMs.

The other two experiments were designed to manipulate the spatio-temporal signature of the jumping jack articulation sequence under the masking, particularly the spatio-temporal features leading to extrema in global shape appearance. In Experiment III, the PLS pattern with the adapted mask are manipulated so that the hands follow circular movements. As a consequence, the lateral extension is reduced, however, the occurrence of LOMs is increased. In other words, the strength of the salient extremal features for global discriminating shape features is diminished. At the same time the frequency and integrated strength of LOM is increased again. We expected that now with the elimination of the global pattern extrema the descrimination rate is significantly reduced. The observed effect was less strong than expected. We conclude that the discriminability is somewhat restored by the increase in LOM patterns. We should not, however, draw any conclusions about the relative weights of the perceptual contribution of the local and the global features.

Experiment IV was finally designed to systematically reduce the variance of the pattern of lateral body pose extension. By shifting the temporal movement phases of the left body parts over the whole motion sequence the lateral extension of the global appearance pattern remains almost constant. At the same time, though, the occurrence of LOMs is diminished. In the discrimination experiments observers identified distinct temporal events which were characterized by longitudinal extensions of the global appearance pattern. In terms of causal relations between features and the detection of key pose events, we cannot unambiguously identify a conjunction of such properties with the detection of features or extrema in the feature dimensions discussed, such as LOMs or local extrema in motion energy. Such distinct events in an otherwise rather homogeneous spatio-temporal pattern might be identified by extrema in the temporal evolution of configural patterns and their appearance. Here, it would be reasonable to investigate whether such extrema may also be detected and judged as key events for spatio-temporal appearances generated by non-human shapes, such a deformable objects of arbitrary shape.

### 4.3. Features indicating characteristic articulated motion patterns

The observed correlation between the combined lateral and longitudinal extension of the PLS and the human recognition performance might provide an indication on the characteristic properties of salient form features in the context of biological motion perception. While occurrences of a lateral or longitudinal extension seem to have an impact on the perceptual salience of a PLS subsequence, it is the temporal relationship which renders this presence of characteristic form information special and distinctive. We hypothesize that local temporal maxima in (potentially a variety of) feature channels form the basis for an increase in the perceptual salience of a biological motion sequence. In other words, the conjecture advocated here is that first order mechanisms operate upon the feature representations to register local temporal contrasts in the respective feature channels (or combinations of these). They, in turn, generate event-triggers to serve as a marker, e.g., to steer the learning of form and motion pattern characteristics. We do not claim that these feature channels are only to be found in the spatial domain. On the contrary, we are convinced, that dependent on the type of action displayed in the PLS, e.g., patterns of LOM (Casile and Giese, 2005) are candidates to explain an increased perceptual salience just as well and are in line with this hypothesis. It is worth to note that due to the nature of human kinetics, such temporal maxima in feature channel activations co-occur with local extrema in the energy of whole body or limb motions (see Figure 4). Thus, motion energy in turn may be a candidate to tag local maxima of form/motion based feature characteristics.

## Author contributions

GL developed the presented stimulus design and the overall setup of the experiment. He implemented and conducted the experiments together with students, as well as performed the interpretation and statistical analysis of the experimental data. HN developed the overall design of the experiments in collaboration with GL, participated in the design and the conduction of the experiments, as well as in the interpretation of the experimental results.

### Conflict of interest statement

The authors declare that the research was conducted in the absence of any commercial or financial relationships that could be construed as a potential conflict of interest.

## Footnotes

^1^The dataset is made publicly available upon request under http://uulm.de/imagedb

^2^http://psychtoolbox.org

^3^Helmholtzstrae 20, 89081 Ulm, Germany.
